# Value of pretreatment MRI determined parameters for predicting outcome after radio-frequency ablation of hepatocellular carcinoma

**DOI:** 10.1186/1470-7330-15-S1-S7

**Published:** 2015-10-02

**Authors:** R Dresen, K Michielsen, F De Keyzer, C Verslype, B Topal, R Aerts, V Vandecaveye

**Affiliations:** 1 Department of Radiology, Leuven Cancer Institute, University Hospitals Leuven, KU Leuven, Leuven, Belgium; 2 Department of Hepatology, Leuven Cancer Institute, University Hospitals Leuven, KU Leuven, Leuven, Belgium; 3 Department of Surgery, Leuven Cancer Institute, University Hospitals Leuven, KU Leuven, Leuven, Belgium

## Aim

To evaluate whether pretreatment magnetic resonance imaging (MRI) determined imaging parameters are predictive for outcome in hepatocellular carcinoma (HCC) treated with radio-frequency ablation (RFA).

## Methods

Thirty-seven patients with HCC treated by RFA were evaluated. Lesion number, size and segmental location, T2-weighted (w), arterial, portal-venous and venous contrast-phase, b600 diffusion-w imaging (DWI) and delayed phase contrast-enhanced imaging pattern were assessed at MRI. The separate imaging patterns as well as pretreatment clinical variables were correlated with outcome (disease free survival longer or shorter than 1 year) using a chi-square test with multiple variables and Mann-Whitney U test respectively. Pretreatment clinical variables and imaging parameters were correlated with Keratin 19 and microvascular invasion status at the biopsy during RFA.

## Results

None of the pretreatment patient- or tumour-related parameters correlated to disease free survival (p>0.5).

The portal-venous, venous phase and b600 DWI imaging pattern showed strongest correlation with disease free survival (p=0.00023, p=0.00003 and p=0.0002 respectively). Also correlation was found for T2w imaging pattern (p=0.007), and hepatobiliary phase imaging pattern (p=0.017). Patients with tumour recurrence within 1 year (n=14) showed persistent venous rim- or nodular enhancement in 13 patients and b600 DWI rim-like hyperintensity in 9 patients correlating with microvascular invasion at biopsy (p=0.04). Patients disease free for at least 1 year (n=23) showed venous wash-out in 22 of 23 patients and whole-lesion hyperintensity b600 DWI in 18 patients.

## Conclusion

Pretreatment venous rim-enhancement and rim-like intensity at b600 DWI were strongest predictors of treatment failure within the first year after RFA of HCC.

